# Production of wheat bread without preservatives using sourdough starters

**DOI:** 10.1080/13102818.2014.965057

**Published:** 2014-10-30

**Authors:** Rositsa Denkova, Svetla Ilieva, Zapryana Denkova, Ljubka Georgieva, Mariya Yordanova, Dilyana Nikolova, Yana Evstatieva

**Affiliations:** ^a^ Department of Biotechnology, Sofia University ‘St. Kliment Ohridski’, Sofia, Bulgaria; ^b^ Department of Microbiology, University of Food Technologies, Plovdiv, Bulgaria; ^c^ Institute of Cryobiology and Food Technology, Bulgarian Academy of Sciences, Sofia, Bulgaria

**Keywords:** *Lactobacillus*, *Propionibacterium*, sourdough, starter, bread, mold, roping

## Abstract

In order for the beneficial effects of sourdough application in breadmaking to take place a proper selection of lactic acid bacteria species and strains, an appropriate technology and effective control of the purity and activity of the selected cultures. Four symbiotic starters for sourdough for the production of bread were developed and probated in a production laboratory using the selected strains *Lactobacillus brevis* LBRZ7, *L. buchneri* LBRZ6, *L. plantarum* X2, *L. paracasei* RN5, *L. sanfranciscensis* R and *L. fermentum* LBRH10 and the probiotic strain Propionibacterium freudenreichii ssp. shermanii NBIMCC 327. The starter sourdoughs that include *Propionibacterium freudenreichii* ssp. shermanii NBIMCC 327 had greater antimicrobial activity against saprophytic microorganisms: *Bacillus subtilis, B. mesentericus, Aspergillus niger, Penicillium sp*. and *Rhizopus* sp., but none of them inhibited the growth of bakery yeasts *Saccharomyces cerevisiae*. It was established that in order to prevent bacterial spoilage 10% of the selected starter sourdoughs had to be added in the breadmaking process, while for prevention of mold spoilage the necessary amount of starter sourdough had to be between 15% and 20%.The application of the developed starters for the production of wheat bread guarantees longer shelf life and no adverse alterations in the features of the final bread.

## Introduction

According to the European Commission Concerted Action on Functional Food Science in Europe (FuFoSE) and the International Life Sciences Institute (ILSI), Europe ‘a food that beneficially affects one or more target functions in the body beyond adequate nutritional effects in a way that is relevant to either an improved state of health and well-being and/or reduction of risk of a disease’ is called a functional food.

Not all strains of the genus *Lactobacillus* can be included in the composition of functional foods. For each type of product there are selected strains of microorganisms that carry out their metabolic processes, which contribute to the formation of the flavour and define the terms of storage of the final products.

Bakery products have a very short shelf life. Their quality depends on the time interval between baking and consumption.[1] Spoilage of bakery products is mainly due to the growth of moulds, the main species belonging to the genera *Aspergillus*, *Fusarium* and *Penicillium*, as well as to the roping of the bread, caused by *Bacillus* sp., especially *B. subtilis* and *B. licheniformis*.[2] The freshness of bread depends on the flavour, appearance and crispness of the crust, the hardness of the crumb and the volume of the bread. The taste of the bread, however, is considered the most important feature for consumers, as a criterion for eligibility of the products.[3] During storage, reduction in the freshness of the bread along with the increase of the hardness of the crumb result in loss of acceptable appearance to consumers, a process known as staling.[4]

There are several physical methods for food preservation: heat treatment, cold storage, modified atmosphere storage, drying and freeze-drying.[5] The protection of bakery goods from mould spoilage is achieved primarily through inactivation of contaminating spores using (1) IR and microwave radiation; (2) fungal inhibitors such as ethanol and propionic, sorbic, benzoic and acetic acid and their salts; (3) appropriate packaging techniques such as modified atmosphere packaging; and (4) addition of sourdough.[6–8]

The addition of sourdough is the best technique to keep the bread from spoilage meeting consumer's demand for natural food without additives.[7] Sourdough is a mixture of flour (from wheat, rye, rice, etc.) and water, which is fermented by the action of lactic acid bacteria and yeasts.[7] These microorganisms usually come from flour, dough ingredients or the environment.

The objectives to be achieved by the use of sourdough are a significant increase in the shelf life and the nutritional value of bread and improvement of the organoleptic properties of bread. The increase in the retention time of the sourdough bread is due to the higher levels of acidity and the higher concentration of produced organic acids in comparison to the commercial bread produced by using only yeasts. Moreover, the addition of sourdough increases the bioavailability of minerals in bread as a result of phytate hydrolysis. The improvement of the organoleptic properties of bread is due to the presence of non-volatile and volatile compounds that improve the flavour of the bread. However, the production of bread using sourdough is a very sensitive method that depends on various parameters which need to be controlled. The most important parameters of fermentation are pH during fermentation, the temperature of fermentation and the careful selection of starter cultures for obtaining sourdough with specific and desired properties.[3,9]

There are a number of benefits of the application of sourdough in bread making: improvements in the volume of the bread and the structure of the crumb,[10,11] the flavour,[12] the nutritional value [13,14] and shelf life,[1,15–18] due to the delay of the process of staling and the prevention of mould and bacterial spoilage.[19,20] These positive effects are associated with the metabolic activity of the selected pure cultures of yeast and homo- and heterofermentative lactic acid bacteria in the composition of the sourdough, e.g. lactic acid fermentation, proteolysis, exopolysaccharide production and synthesis of volatile and antimicrobial compounds.[1,7,21] The fermentation of sourdough can affect intestinal health through several mechanisms: (1) modulation of the complex dietary fibres and the subsequent pattern of fermentation, (2) production exopolysaccharides with prebiotic properties, and (3) possible transfer of metabolites from the fermentation of lactic acid bacteria that affect the intestinal microflora.[22] In order for those beneficial effects to take place a proper selection of lactic acid bacteria species and strains, an appropriate technology and effective control of the purity and activity of the cultures are required. The selection of pure cultures consists of using a species or a combination of species specific to the technological process, fully adapted to the environment of sourdough and to the applied fermentation conditions.[23,24]

There is a considerable diversity of lactic acid bacteria isolated from sourdough: *L. acidophilus*, *L. delbrueckii*, *L. farciminis* (obligate homofermentative), *L. plantarum*, *L. homohiochii*, (facultative heterofermentative), *L. brevis*, *L. buchneri*, *L. fermentum*, *L. hilgardii*, *L. sanfranciscensis*, *L. viridiscens*, *L. panis and L. pontis* (obligate heterofermentative).[7,25,26]

With the inclusion of starter cultures, the pH falls very quickly, so the whole manufacturing process is accelerated, which leads to economic benefits for the producer. The secondary effects of the acidification and the acceleration of the fermentation time include changes in the activity of the enzymes of the cereal substrates or the bacterial strains.[1] The greater part of the starter cultures are natural isolates of the desired microorganisms normally found in cereal substrates.[27,28]

The aim of the present study was to develop starter symbiotic combinations for wheat sourdough and to determine the percentage of starter sourdough to be used in bread making that would extend the shelf life of bread without preservatives without any adverse alterations of bread quality.

## Materials and methods

### Microorganisms

The studies in this work were conducted using six strains of the genus *Lactobacillus*, isolated by the authors and currently included in the collection of microorganisms of Department ‘Microbiology’ at the University of Food Technologies, Plovdiv, Bulgaria and *Propionibacterium freudenreichii* ssp. *shermanii* NBIMCC 327. The six strains were as follows: *Lactobacillus brevis* LBRZ7 and *L. buchneri* LBRZ6 (isolated from fermented cabbage), *L. plantarum* X2, *L. paracasei* RN5 and *L. sanfranciscensis* R (isolated from naturally fermented sourdough), *L. fermentum* LBRH10 (of human origin) (unpublished data). These strains had been identified by physiological, biochemical and molecular-genetic methods.[29,30]

### Media

Sterile skimmed milk with titratable acidity 16–18°T (Scharlau)

MRS-broth (medium of Man, Rogosa, Sharpe) (Scharlau)

MRS-agar. Composition (g/dm^3^): MRS-broth (Scharlau), agar – 20.

LAPTg10-broth. Composition (g/dm^3^): peptone – 15, yeast extract – 10; tryptone – 10, glucose – 10. pH is adjusted to 6.6–6.8 and Tween 80–1 cm^3^/dm^3^ is added.

LAPTg10-agar. Composition (g/dm^3^): LAPTg10-broth medium, agar – 20.

Elective medium for *Propionibacterium* sp. Composition (g/dm^3^): tryptone – 10, yeast extract – 10; Na-lactate (fresh) – 10 cm^3^, KH_2_PO_4_ – 2.5, MnSO_4_ – 0.005, agar – 15; pH is adjusted to 6.8. [* Na-lactate (fresh) – 7 g of Na-lactate lactic acid is neutralized with 3.1 g NaOH crystals and after that the remaining salts dissolved in distilled water are added.]

Saline solution. Composition (g/dm^3^): NaCl – 5.

### Cultivation and storage of the studied microorganisms

The studied strains of microorganisms were cultured in a liquid medium (MRS-broth) and on agar medium (MRS-agar) at 30 °C for *L. brevis* LBRZ7, *L. buchneri* LBRZ6 and *L. sanfranciscensis* R and *L. plantarum* X2, and at 37 ºC for *L. paracasei* RN5 and *L. fermentum* LBRH10 for different periods of time. *Propionibacterium freudenreichii* ssp. *shermanii* NBIMCC 327 was cultured in skimmed milk at 30 °C. All tested *Lactobacillus* strains had been isolated from a single colony and were cultured in MRS-broth medium for 24 hours.

### Development of starters for sourdough for wheat and rye bread

First, the Basic Combination, containing *L. paracasei* RN5, *L. plantarum* X2, *L. brevis* LBRZ7 and *L. fermentum* LBRH10 in a ratio of 1:2:1:1 was prepared.

Then the four new combinations were prepared as follows:

Combination 1 – Basic Combination:*L. sanfranciscensis* LSR = 2:1;
Combination 2 – Basic Combination:*L. sanfranciscensis* LSR:*P. frendenreichii* ssp. *shermanii* NBIMCC 327 = 2:1:1;
Combination 3 – Basic Combination:*L. buchneri* LBRZ6 = 2:1;
Combination 4 – Basic Combination:*L. buchneri* LBRZ6:*P. frendenreichii* ssp. *shermanii* NBIMCC 327 = 2:1:1.

The four new combinations were subcultured in MRS-broth daily for the duration of 96 hours and the changes in the titratable acidity and the number of viable cells of the strains of *Lactobacillus* sp. and *Propionibacterium* sp. were monitored. The LAB counts were determined by appropriate 10-fold dilutions and spread plating on coloured LAPTg10-agar medium. The number of propionic acid bacteria was determined by appropriate 10-fold dilutions and pour plating on elective medium for *Propionibacterium* sp. A standard method [31] was used for measurement of the titratable acidity.

### Preparation of cellular suspensions for the inoculation of flour/water mixture

For the production of sourdough with the four developed multistrain starters the 24-hour cultural suspensions of the *Lactobacillus* strains, included in the starter, were mixed according to the proportions given in ‘Development of starters for sourdough for wheat and rye bread’ and homogenized. The lactobacilli cells were harvested by centrifugation at 5000 ×*g* for 15 minutes, washed twice with PBS-buffer and the biomass sludge was resuspended to the initial mixed suspension volume with sterile saline solution. Then the cellular suspension was mixed in the respective ratio with the 24–48 hour cultural suspension of *P. freudenreichii* ssp. *shermanii* NBIMCC 327 in milk and the obtained homogenized mixture was used for inoculation of the flour/water mixture for the production of multistrain sourdough.

### Preparation of sourdoughs with multistrain starters

The cellular suspensions of the four combinations were obtained using the procedure described above and used to inoculate the flour/water mixtures. The changes in the concentration of viable cells of lactic acid bacteria, yeasts and moulds and in the titratable acidity of the sourdoughs were monitored by passaging every 24 hours over a period of 96 h of cultivation at 30 ºC according to the following scheme:

1. first day – 44% flour: 56% tap water and 10% of the mixed cellular suspension;
2. second to fifth day: 25% sourdough from the previous day: 75% fresh mixture flour/water. The fresh mixture was prepared in a ratio: 44% flour/ 56% water.

The number of lactic acid bacteria, propionic acid bacteria, yeasts and moulds was determined by appropriate 10-fold dillusions and spread plating on coloured LAPTg10-agar medium for the enumeration of lactic acid bacteria or pour plating on elective medium for *Propionibacterium* sp. for the enumeration of propionic acid bacteria. Total titratable acidity is determined by a standard method.[31]

### Determination of the antimicrobial activity against saprophytic microorganisms

The agar diffusion method was used to determine the antimicrobial activity of the four prepared starter sourdoughs. A dilution in a ratio of 1:1 of a sourdough:saline solution of each of the sourdoughs was prepared. The antimicrobial activity was tested against the following saprophytic test microorganisms: bacteria – *B. subtilis*, *B. mesentericus*; yeasts – *Saccharomyces cerevisiae*, moulds – *Aspergillus niger*, *Penicillium* sp., *Rhizopus* sp. A suspension of each of the test microorganisms (10^6^–10^7^ CFU/cm^3^) was used to inoculate a Petri dish with agar medium and after the hardening of the agar wells (7 mm) were prepared. 0.06 cm^3^ of the dillusions were pipetted in the wells of the plates and the plates with the test microorganisms were incubated at 37 °C for 24–48 hours, and then the inhibition zones in mm were reported.

### Approbation of the starter sourdoughs in the production laboratory

‘Mother’ doughs with 10%, 15% or 20% of the 96-hour starter sourdoughs were prepared. Еach dough was prepared with 1.5% NaCl, 2% yeast starter, the respective percentage of sourdough and tap water (the amount of water was determined by the water absorption of the type of flour). The dough was kneaded in a mixer: slow kneading (1000 rpm) for 4 min and fast kneading (1400 rpm) for 10 min, after which the dough was rested for about 10 min in a proofer in order for its elastic properties to be improved. Loaves were formed and placed in the forms. Then followed leavening for about 40–45 min at 30 °C and 80 ± 5 RH. In the production laboratory wheat bread with sourdough as well as control bread (bread without sourdough with starter) were baked, cooled and evaluated. Baking was carried out at 225 ± 5 °C for 30 min in a deck oven. Loaves were allowed to cool for 120 min at room temperature.

### Determination of bacterial spoilage of baked bread

The determination of the appearance of bacterial spoilage was performed by 10 judges in the production laboratory and was evaluated according to a scale of I–IV, with each of the degrees corresponding to the following descriptions:

- barely noticeable (pleasant fruity odour);
- weak (change in the odour – distinct);
- medium (moisty, sticky crumb, sharp odour);
- strong (unpleasant odour, brown-yellow crumb).

### Determination of mould spoilage of baked bread

The determination of the appearance of mould spoilage was performed by 10 judges in the production laboratory and was evaluated according to the appearance of single mould colonies.

## Results and discussion

### Selection of strains

In order to be included in the composition of the starters for sourdough for bread a mandatory selection lactic acid bacteria strains is required. It is crucial to select lactobacilli strains that accumulate high concentrations of viable cells in short time in order for a targeted fermentation process to be conducted. Therefore, the reproduction ability of the lactobacilli strains and the produced amount of lactic or lactic and acetic acids were examined. The ability of the strains *L. paracasei* RN5, *L. plantarum* X2, *L. brevis* LBRZ7, *L. fermentum* LBRH10, *L. buchneri* LBRZ6 and *L. sanfranciscensis* LSR to grow in flour/water environment and to accumulate high concentrations of viable cells and acids was examined. The selected lactobacilli strains grew very well in the flour/water environment, reaching 10^14^–10^15^ CFU/cm^3^ viable cells by the 96th hour and the acidity of the resulting sourdoughs increased to values above 10 °N.[32]

### Development of symbiotic combinations

Based on the results in our previous studies (unpublished data) on starter cultures for sourdough for wheat and rye bread, the starter with the best performance was determined to be the combination of *L. paracasei* RN5:*L. plantarum* X2:*L. brevis* LBRZ7:*L. fermentum* LBRH10 in a ratio of 1:2:1:1 (called Basic Combination). Four novel combinations – two, in which the *L. buchneri* LBRZ6 was included (for wheat bread), and two, in which *L. sanfranciscensis* LSR was included (for rye bread), were designed.

Combination 1 – Basic Combination:*L. sanfranciscensis* LSR = 2:1;
Combination 2 – Basic Combination:*L. sanfranciscensis* LSR:*P. frendenreichii* ssp. *shermanii* NBIMCC 327 = 2:1:1;
Combination 3 – Basic Combination:*L. buchneri* LBRZ6 = 2:1;
Combination 4 – Basic Combination:*L. buchneri* LBRZ6:*Propionibacterium frendenreichii* ssp. *shermanii* NBIMCC 327 = 2:1:1.

**Figure 1.**
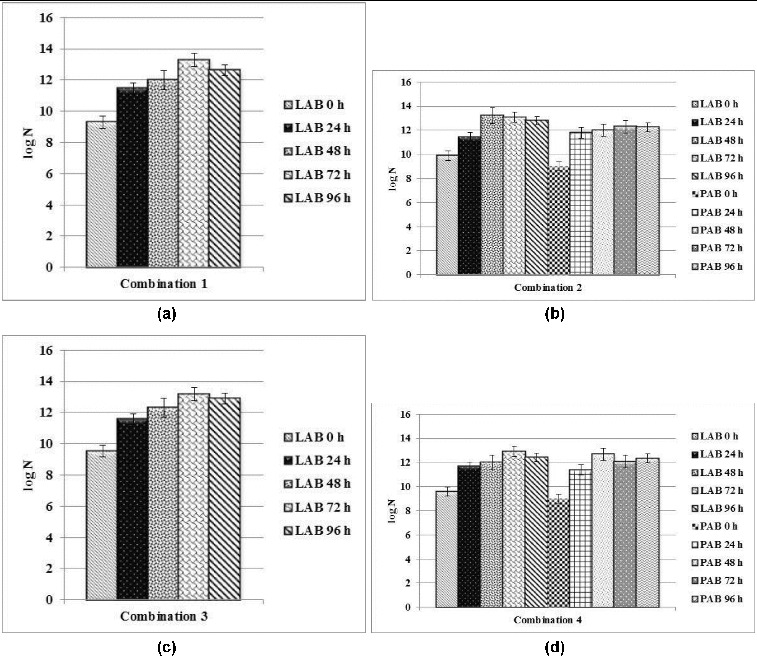
Changes in the concentration of viable cells of lactic acid bacteria (LAB) and propionic acid bacteria (PAB) in the four new combinations subcultured in MRS-broth every 24 hours for the duration of 96 hours: (a) Combination 1, (b) Combination 2, (c) Combination 3 and (d) Combination 4.

The four new combinations were subcultured every 24 hours for the duration of 96 hours. The change in the titratable acidity and the concentration of viable cells of lactobacilli and propionic acid bacteria in passaging was monitored (Figure 1(a)–(d), Table 1).

**Table 1.** Changes in the titratable acidity of the four new combinations subcultured in MRS-broth every 24 hours for the duration of 96 hours.

	0 h	24 h	48 h	72 h	96 h
Combination 1	69.9 ± 1.0	205.6 ± 1.1	260.0 ± 2	289.0 ± 1.9	218.0 ± 1.8
Combination 2	71.7 ± 1.2	206.5 ± 1.0	267.5 ± 1.7	270.0 ± 1.8	214.0 ± 1.1
Combination 3	69.4 ± 0.8	224.0 ± 1.4	275.5 ± 1.1	276.0 ± 2.1	206.0 ± 1.5
Combination 4	69.9 ± 1.6	232.9 ± 2.0	276.8 ± 1.8	275.0 ± 2.3	210.0 ± 1.0

The results for the titratable acidity and the concentration of viable cells upon passaging clearly indicated a symbiotic relationship between the strains in the combinations.

### Approbation of starter cultures for wheat bread in a production laboratory

The four combinations were kneaded every 24 hours for the duration of 96 hours in a production laboratory. The changes in the acidity and the aroma of the sourdoughs in passaging as well as the concentration of viable cells of the *Lactobacillus* strains at the 0th and at the 96th hour were determined. The results of these studies are summarized in Table 2.

**Table 2.** Dynamics of the change in the acidity, aroma and concentration of viable cells in the process of passaging of the sourdoughs every 24 hours for 96 hours.

	0 h	24 h		48 h		72 h		96 h		
	*N* (CFU/cm^3^)	TTA (°N)	Aroma	TTA (°N)	Aroma	TTA (°N)	Aroma	TTA (°N)	Aroma	*N* (CFU/cm^3^)
1	4.4 × 10^8^	15.2	All sourdoughs have the same aroma.	13.0	The type of the aroma is the same in all four	14.8	The acid hint in the aroma can be sensed in all	15.4	The type of the aroma is the same in all four	3.0 × 10^10^
2	4.6 × 10^7^	154	Sourdoughs with Starters 2 and 4 have sweetish odour.	14.6	sourdoughs. Sourdoughs with Starters 1 and 3 have	14.4	sourdoughs, but the sourdough with Starter 4 has less acidic odour.	13.6	sourdoughs. Sourdoughs with Starters 1 and 3 have	1.1 × 10^11^
3	3.9 × 10^7^	13.0	Sourdoughs with Starters 5 and 7 are with sour aroma. The texture of all	13.0	milder aroma. Sourdoughs with Starters 2 and 4 have slightly pungent	14.6		14.8	sourer aroma. The sourdoughs with Starters 2 and 4 have not so sharp, but softer	6.0 × 10^13^
4	5.5 × 10^8^	13.8	sourdoughs is washy, which is typical for the beginning of the process.	12.8	aroma.	13.8		15.0	aroma.	7.0 × 10^11^

Note: TTA – total titratable acidity.

For 96 hours of passaging in the production laboratory it was clear that the sourdoughs with the new starters were stabilized in terms of acidity. The biggest change in the concentration of viable cells was observed in Starter 3 (5 logN), followed by Starter 2 (4 logN), Starter 4 (3 logN) and Starter 1 (2 logN).

Each of the four 96-hour starter sourdoughs was diluted in saline solution in a ratio of 1:1 and the prepared diluted suspension was used to determine the antimicrobial activity of the sourdoughs against saprophytic microorganisms (Table 3). All sourdoughs had inhibitory activity against *B. subtilis*, *B. mesentericus*, *A. niger*, *Rhizopus* sp. and *Penicillium* sp. Neither of the four sourdoughs inhibit the bakery yeast strain *Saccharomyces cerevisiae*. A comparison between the starters with *L. buchneri* LBRZ6 (Starter 3 and Starter 4) showed that Starter 4 (with the participation of *P. freudenreichii* ssp. *shermanii* NBIMCC 327) had higher antimicrobial activity against the saprophytes included in the study. The similar conclusions could be drawn from the comparison between Starter 1 and Starter 2, the two starters with *L. sanfranciscensis* LSR, which was clear evidence of the inhibiting effect of *P. freudenreichii* ssp. *shermanii* NBIMCC 327 against saprophytes.

**Table 3.** Antimicrobial activity of the four starter sourdoughs at the 96th hour at a dilution of 1:1 against saprophytic microorganisms. The values are in mm. Diameter of the well: 7 mm.

	Starter No.			
Saprophyte	1	2	3	4
*Bacillus subtilis*				
7.25 ×10^5^ CFU/cm^3^	10	14	13	14.5
*Bacillus mesentericus*				
5.5 × 10^5^ CFU/cm^3^	17	19	12	14
*Aspergillus niger*				
2 × 10^4^ CFU/cm^3^	–	10	10	14
*Saccharomyces cerevisiae*				
4.25 × 10^5^ CFU/cm^3^	–	–	–	–
*Penicillium* sp.				
9 × 10^5^ CFU/cm^3^	9	12	10	12
*Rhizopus* sp.				
1.4 × 10^4^ CFU/cm^3^	15	18	14.5	19

Bread was baked with different percentage of the four sourdoughs (10%; 15% and 20%) with the four 96-hour starter sourdoughs in order to determine the best starter combination for wheat and rye bread, as well as the optimum percentage of starter sourdough to be incorporated in the preparation of the ‘mother’ dough to prevent mould and *Bacillus* growth without adversely affecting the organoleptic characteristics of the final bread.

Highly contaminated flours containing high concentration of *Bacillus* spores (over 10^2^ CFU/g) were used for the determination of bacterial and mould spoilage of the baked breads. The baked breads with 10%; 15% or 20% of the 96-hour starter sourdoughs were incubated in non-aseptic conditions in parallel experiments at room temperature and in a thermostat at 37 °C for 96 hours for the determination of bacterial spoilage and for 120 hours at 30 °C and at room temperature for determination of mould spoilage.

It was established that bacterial spoilage due to the growth of representatives of the genus *Bacillus* occurred earlier in the control loaf incubated at 37 °C, than in that incubated at room temperature. The earliest appearance of bacterial spoilage was in the control bread – on the 48th hour after taking the loaves out of the oven at 37 °C and on the 72nd hour at room temperature. According to the standard requirements there should be no signs of bacterial decay up to the 48th hour. The control bread did not meet the standard requirements for microbial safety of bakery products. Upon addition of 10% of sourdough there were signs of bacterial spoilage on the 72nd hour both at 37 °C and at room temperature. If the percentage of sourdough addition rose to 15%, in the obtained sourdough bread with Starter 1 or Starter 3 bacterial decay became visible on the 96th hour, while variants baked with sourdough with Starter 2 or Starter 4 (they both included the probiotic strain *P. freudenreichii* ssp. *shermanii* NBIMCC 327) showed no signs of bacterial spoilage even on the 96th hour both at 37 °C and at room temperature. Upon addition of 20% of sourdough no bacterial spoilage was established except for the variants prepared with 20% of sourdough with Starter 1 or Starter 3 and were stored at 37 °C (Table 4).

**Table 4.** Bacterial bread spoilage by *Bacillus* sp. during incubation of the baked breads at 37 °C and at room temperature.

		24 h		48 h		72 h		96 h	
Variants	Temperature	DS	Aroma	DS	Aroma	DS	Aroma	DS	Aroma
Control	37 °C	–	No	I	Yes	II	Yes	III	Yes
	Room temperature	–	No	–	No	I	Yes	II	Yes
Combination 1; 10%	37 °C	–	No	–	No	I	Yes	II	Yes
	Room temperature	–	No	–	No	I	Yes	II	Yes
Combination 1; 15%	37 °C	–	No	–	No	–	No	I	Yes
	Room temperature	–	No	–	No	–	No	I	Yes
Combination 1; 20%	37 °C	–	No	–	No	–	No	I	Yes
	Room temperature	–	No	–	No	–	No	–	No
Combination 2; 10%	37 °C	–	No	–	No	I	Yes	II	Yes
	Room temperature	–	No	–	No	I	Yes	II	Yes
Combination 2; 15%	37 °C	–	No	–	No	–	No	–	No
	Room temperature	–	No	–	No	–	No	–	No
Combination 2; 20%	37 °C	–	No	–	No	–	No	–	No
	Room temperature	–	No	–	No	–	No	–	No
Combination 3; 10%	37 °C	–	No	–	No	I	Yes	II	Yes
	Room temperature	–	No	–	No	I	Yes	II	Yes
Combination 3; 15%	37 °C	–	No	–	No	–	No	I	Yes
	Room temperature	–	No	–	No	–	No	I	Yes
Combination 3; 20%	37 °C	–	No	–	No	–	No	I	Yes
	Room temperature	–	No	–	No	–	No	I	Yes
Combination 4; 10%	37 °C	–	No	–	No	I	Yes	II	Yes
	Room temperature	–	No	–	No	–	No	–	No
Combination 4; 15%	37 °C	–	No	–	No	–	No	–	No
	Room temperature	–	No	–	No	–	No	–	No
Combination 4; 20%	37 °C	–	No	–	No	–	No	–	No
	Room temperature	–	No	–	No	–	No	–	No

Note: I – barely noticeable (pleasant fruity odour).

II – weak (change in the odour – distinct).

III – medium (moisty, sticky crumb, sharp odour).

IV – strong (unpleasant odour, brown-yellow crumb).

DS = degree of spoilage.

The earliest appearance of mould spoilage was in the control bread – on the 72nd hour both at 30 °C and at room temperature. Upon addition of 10% of sourdough the first signs of spoilage became noticeable on the 96th hour, the degree of mould spoilage being the least in the variants made by the inclusion of sourdough with Starter 2 or Starter 4 both at 30 °C and at room temperature. If the percentage of incorporation of starter sourdough in the bread-making process was increased to 15%, mould spoilage became visible on the 120th hour in all variants both at 30 °C and at room temperature, except for those prepared with sourdough with Starter 2 or Starter 4 (they both included the probiotic strain *P. freudenreichii* ssp. *shermanii* NBIMCC 327) that were stored at room temperature in which there was no mould spoilage even on the 120th hour. Upon addition of 20% of sourdough there was no mould spoilage up to the 120th hour (Table 5).

**Table 5.** Mould bread spoilage during incubation of the baked breads at 37 °C and at room temperature.

Variants	Temperature	24 h	48 h	72 h	96 h	120 h
Control	30 °C	No	No	Yes	Yes	Yes
	Room temperature	No	No	Yes	Yes	Yes
Combination 1; 10%	30 °C	No	No	No	Yes	Yes
	Room temperature	No	No	No	Yes	Yes
Combination 1; 15%	30 °C	No	No	No	No	Yes
	Room temperature	No	No	No	No	Yes
Combination 1; 20%	30 °C	No	No	No	No	No
	Room temperature	No	No	No	No	No
Combination 2; 10%	30 °C	No	No	No	Yes/No – single colonies	Yes
	Room temperature	No	No	No	Yes/No – single colonies	Yes
Combination 2; 15%	30 °C	No	No	No	No	Yes
	Room temperature	No	No	No	No	No
Combination 2; 20%	30 °C	No	No	No	No	No
	Room temperature	No	No	No	No	No
Combination 3; 10%	30 °C	No	No	No	Yes	Yes
	Room temperature	No	No	No	Yes	Yes
Combination 3; 15%	30 °C	No	No	No	No	Yes
	Room temperature	No	No	No	No	Yes
Combination 3; 20%	30 °C	No	No	No	No	No
	Room temperature	No	No	No	No	No
Combination 4; 10%	30 °C	No	No	No	Yes/No – single colonies	Yes
	Room temperature	No	No	No	Yes/No – single colonies	Yes
Combination 4; 15%	30 °C	No	No	No	No	Yes
	Room temperature	No	No	No	No	No
Combination 4; 20%	30 °C	No	No	No	No	No
	Room temperature	No	No	No	No	No

Experimental data suggest accelerating of the fermentation. The doughs obtained applying the new starters were tougher, more elastic, and the pieces of bread are higher. As in previous studies with sourdough, the quality of wheat bread was improved by the addition of sourdough. Addition of sourdoughs yielded breads with greater specific loaf volumes when compared to the control. Crumb firmness values showed the opposite trend, with sourdough breads showing a softer and lighter crumb than the control, which was in compliance with previously published studies.[33–35] The results confirmed the results obtained by Clarke and Arendt [36] demonstrating that the metabolic products of the yeasts and LAB improve the properties of the flour, as well as aroma, taste, nutritive value and shelf life of the bread.

The incorporation of 15% or more sourdough in the bread-making process inhibited the growth of both bacterial and mould spores and ensured long shelf life of the baked bread,[2] and while the increase in acidification might be necessary for the optimal swelling and baking of bread, for the control of enzymatic activities, elasticity and suitability of the crumb, and for prolonging shelf life [37]; in contrast, excessive acidification has a deleterious effect on some rheological parameters.[37,38] The sourdoughs with the developed starters, described in the present article, were incorporated in a ratio of 15% in non-sterile (non-aseptic) conditions, in contrast to the experiments, described by Mentes et al. [2] which were conducted in aseptic conditions. The prevention of bacterial and mould spoilage during incubation of the baked bread at room temperature (25 – 30 °C) was also demonstrated in the present article.

Currently the two starter sourdoughs (sourdough with Starter 2 for rye bread and sourdough with Starter 4 for wheat bread) are still being passaged daily in the production laboratory.

## Conclusion

Four symbiotic starter combinations were developed and probated in a production laboratory using the selected *Lactobacillus* strains that were able to grow in flour/water environment accumulating high concentrations of viable cells: *L. brevis* LBRZ7, *L. buchneri* LBRZ6, *L. plantarum* X2, *L. paracasei* RN5, *L. sanfranciscensis* R and *L. fermentum* LBRH10 and the probiotic propionic acid bacterial strain *P. freudenreichii* ssp. *shermanii* NBIMCC 327. It was established that in order to prevent bacterial spoilage 10% of the selected starter sourdoughs (sourdough with Starter 2 for rye bread and sourdough with Starter 4 for wheat bread, both starters including *P. freudenreichii* ssp. *shermanii* NBIMCC 327) had to be added in the bread-making process while for prevention of mould spoilage the necessary amount of starter sourdough was between 15% and 20%. The developed sourdoughs would allow consumers to prepare and consume delicious and safe bread with extended shelf life without the addition of preservatives.
